# Editorial: Environmental stressors and aquatic animal immune system function

**DOI:** 10.3389/fimmu.2025.1610969

**Published:** 2025-07-01

**Authors:** Christopher J. Martyniuk, Erchao Li, Matthew L. Rise

**Affiliations:** ^1^ Department of Physiological Sciences, College of Veterinary Medicine, University of Florida, Gainesville, FL, United States; ^2^ Laboratory of Aquaculture Nutrition and Environmental Health, School of Life Sciences, East China Normal University, Shanghai, China; ^3^ Department of Ocean Sciences, Memorial University of Newfoundland, St. John’s, NL, Canada

**Keywords:** immune, aquaculture, environment, stressor, infection

Organisms in marine and freshwater environments can be negatively impacted by both natural and anthropogenic stressors. These stressors include extreme weather events due to climate change, fluctuations in water and atmospheric temperature, hypoxic and anoxic conditions, and chemical pollution. Stressors are mitigated via molecular and physiological compensation to prevent adverse outcomes. The immune system is sensitive to both acute and chronic exposures to environmental stressors, which can lead to autoimmune disorders, chronic inflammation, and a weakened immune system. Hence, this may render some individuals more susceptible to infection or coinfection with pathogens and parasites, contributing to higher infection and disease levels relative to unexposed animals.

Understanding how aquatic organisms adjust their immune systems to cope with increased environmental pressure has significant implications for the conservation of species and aquaculture. This is especially important in the face of climate change, which causes shifts in environmental factors such as temperature, oxygen concentration, pH, and salinity, leading to higher mortality events of aquatic species. This Research Topic highlights research that investigates the causes, mechanisms, and mitigation of environmental stressors impacting the immune systems of aquatic animals, specifically nitrogen stress and hypoxia.

This Research Topic contains studies on both crustaceans and fish, underscoring the diversity and complexity of their immune systems. In their review article, Betancourt et al. detail current knowledge of the crustacean immune system, a taxa that is continuously surrounded by pathogens in water and a group subjected to diverse environmental perturbations that can compromise or weaken immune defenses; as the review points out, both abiotic and biotic stressors have driven adaptations in the crustacean immune system. The authors provide a comprehensive overview of key components of the crustacean’s immune system and detail pathogen-associated molecular patterns (PAMPs), highlighting what is known about pattern recognition proteins like b-1,3-glucanase-related proteins and galectin. The review points out that the toll-like receptors (TLRs) are some of the most widely studied receptors in crustaceans, especially in decapods and describes the immune deficiency (IMD) signaling pathway, responsible for stimulating production of antimicrobial peptides (AMPs). Their review points out that further research is needed to understand immunological memory, immune system priming, and integration of immune signaling cascades in crustaceans in the context of environmental factors like pollution, dissolved oxygen, temperature, salinity, and ammonia and how this contributes to disease susceptibility and resistance.

Nitrogen stress involves the accumulation of ammonia and nitrogen in the water, resulting in toxicity and increased mortality events. In their contribution, Luo et al. aimed to elucidate how ammonia-nitrogen (ammonia-N) stress affects the black tiger shrimp *Penaeus monodon* by integrating biochemical, histological, transcriptomic, and metabolomic analyses. Researchers exposed shrimp to ammonia-N for 12 and 96 hours, then assessed immune markers, oxidative stress indicators, and tissue integrity. They observed marked hepatopancreas damage, including atrophy and deformation of lobules, alongside altered enzyme activities linked to oxidative stress and apoptosis. Transcriptomic analysis revealed significant changes in genes related to innate immunity, apoptosis, and metabolic processes, while metabolomic data underscored disruptions in nucleotide turnover, antioxidant defenses, and core metabolic pathways. Collectively, these findings underscore that ammonia-N stress triggers a multifaceted response—encompassing immune activation, oxidative imbalance, and metabolic dysregulation—that compromises shrimp health. From a comparative immunology perspective, the work provides important insights into crustacean immune pathways under environmental stress and offers potential biomarkers for mitigating ammonia toxicity.

Hypoxia, referring to a state of low dissolved oxygen, can have detrimental effects on the physiology of aquatic species, negatively affecting growth, reproduction and behavior. Xing et al. studied the transcriptional response to low oxygen environments in the gill of Crucian carp (*Carassius auratus*). The authors treated the carp to either a normoxic environment (DO level of 6.6 ± 0.3 mg/L) or hypoxic environment (DO level of 0.6 ± 0.3 mg/L) at 22.5 ± 0.5°C) and conducted transcriptomics in the gills. Several hypoxia-related transcripts were identified using predicted Bayesian network analysis and processes affected by low oxygen included glutathione metabolism, nucleotide metabolism, and p53 signaling pathway, among others. Transcriptome data also identified significant changes in genes related to immune defense and energy metabolism, suggesting there are dramatic changes in energy allocation in the gill. More specifically, RIG-I-like receptor signaling pathway and toll-like receptor signaling were important responses during hypoxia, mechanisms potentially underlying reported cell necrosis and inflammation. Taken together, the study demonstrates that low oxygen environments can have implications for immune system function and inflammation, increasing risk of infection or disease during fish production.

Since farmed Atlantic salmon (*Salmo salar*) are increasingly experiencing chronic hypoxia (particularly in summer) due to climate change, it is important to understand the influence of this environmental challenge on innate and adaptive immune system function. Rojas et al. investigated this by: exposing post-smolt Atlantic salmon to hypoxia [40% dissolved oxygen (DO)] versus normoxia (100% DO) for 6 weeks, followed by the intraperitoneal (IP) injection of bacterin (formalin-killed *Aeromonas salmonicida*) or phosphate-buffered saline (PBS control); and 2) sampling head kidney (HK) and blood before IP injection to assess impact of chronic hypoxia on basal immune function, and 6 hours (HK) and 24 hours (HK and blood) post-injection to assess the influence of chronic hypoxia on the innate immune response to this bacterin. The remaining fish were given an IP boost (bacterin or PBS) 4 weeks after the initial injection, and blood was sampled 4 weeks later to assess the impact of chronic hypoxia on IgM titre. Immune parameters assessed included leukocyte respiratory burst (RB), plasma lysozyme concentration, blood cell counts using flow cytometry, and gene expression in the head kidney using qPCR. Chronic hypoxia significantly increased basal (i.e., pre-injection) leukocyte RB activity, plasma lysozyme concentration, the percentage of circulating monocytes and granulocytes, and transcript expression levels of several antibacterial biomarker genes (e.g., *il8a*, *stlr5*). Bacterin stimulation under chronic hypoxia was associated with significant decreases in RB and other immune parameters as compared to the normoxic group (e.g., the expression of antibacterial biomarker genes including *il8a* and *stlr5*). However, the authors did not observe significant hypoxia-associated changes in basal or post-bacterin stimulated serum IgM levels. These discoveries regarding the impact of chronic hypoxia on salmon immune system function (i.e. improved basal innate immune parameters, decreased antibacterial innate immune response, and no effect on serum IgM titers) are important for fish comparative immunology and aquaculture research in the current era of accelerated climate change.

In summary, the immune system in aquatic animal species plays an integral role in protection against bacterial, viral, and other pathogens. However, as we have learned through this Research Topic and other studies, nitrogen stress, hypoxia, and temperature can compromise the immune system response. Multiple stressors have the potential to negatively affect the immune system of aquatic species, and additional research is required to better understand their scope of impact to improve aquaculture practices and manage/predict disease outbreaks due to climate change.

